# “I had to take a ‘dead year’ to undergo surgeries,” the Lived Experiences of University Students with Sickle Cell Disease, an interpretative phenomenological analysis

**DOI:** 10.21203/rs.3.rs-7300043/v1

**Published:** 2025-08-26

**Authors:** Vivian Nabisere, Active Atukunda, Kevina Tracy Mbabazi, Angella Namujuzi, Eric Katagirya

**Affiliations:** Makerere University; Makerere University; Makerere University; Kabale University; Makerere University

**Keywords:** Sickle cell disease, sickle cell anaemia, coping strategies, stigma, university students, institutional policy, disability, osteonecrosis

## Abstract

**Background:**

Sickle cell disease (SCD) is a hereditary blood disorder characterized by the production of abnormal hemoglobin molecules that cause red blood cells to take on a crescent or sickle shape. SCD causes excruciating pain, leading to hospitalizations and negatively affecting patients’ quality of life. In transitioning to university life, students find themselves in a new environment, with demanding new domestic responsibilities and engaging in academic tasks that are perceived as different and are closely related to future success and dealing with their social life. The experiences of university students with SCD in Uganda have not been investigated. This study aimed to explore the lived experiences of university students with sickle cell disease at Makerere University, Uganda.

**Methods:**

The study employed a qualitative approach. We conducted in-depth interviews with university students living with SCD. A total of 15 individuals were purposively recruited and participated in the study. Interpretative phenomenological analysis was applied to explore the lived experiences of students living with SCD and ascertain important themes.

**Results:**

The average age of participants was 23 years (range 19–26), and the majority (60%) were male. Patient experiences were captured in six overarching themes: academic disruptions, experience of stigmatization, the feeling of missing out, support systems, effective self-management and coping strategies, and being normal.

**Conclusions:**

This study highlights the significant challenges faced by university students with SCD in relation to academic interruptions and social integration due to health complications. Despite these challenges, the resilience and coping strategies exhibited by these students underscore their determination to succeed in their academic pursuits. Universities must recognize the unique needs of this population and implement policies that provide adequate support to ensure that students with SCD can thrive both academically and socially.

## Background

Sickle cell disease describes a group of inherited hemoglobin disorders characterized by a predominance of abnormal sickle hemoglobin in erythrocytes. The most common and severe form of sickle cell disease is sickle cell anaemia, which is caused by the homozygous inheritance of sickle haemoglobin from both parents.(1)

The worldwide estimate for neonates born with sickle cell disease each year is 400000, including 300,000 with sickle cell anaemia.(1) The greatest burden is seen in Sub-Saharan Africa, which accounts for more than 75 percent of all sickle cell disease cases, with this proportion expected to rise by 2050.(2) In Africa, sickle cell disease contributes significantly to mortality in children under the age of five,(3) limiting progress toward the United Nations Sustainable Development Goal 3, Good Health and Well-Being, which includes reducing childhood mortality(4). Uganda has the fifth highest sickle cell burden in Africa, with 13.3% of children having sickle cell trait and 5,000–20,000 babies born with SCD every year, of which 80% die before reaching 5 years of age(5). WHO has also challenged countries in Sub-Saharan Africa to develop national strategies for sickle cell disease that address specific goals, targets, and objectives. Despite this, ministries of health face numerous challenges in developing meaningful interventions, including a lack of accurate data on disease burden and distribution within their respective countries.(6)

The main clinical manifestation of SCD is the “acute painful crisis,” which often requires hospitalization.(7) “Sickle cell crisis” is a term that describes several acute conditions such as the vaso-occlusive crisis (acute painful crisis), aplastic crisis, splenic sequestration crisis, hyperhemolytic crisis, hepatic crisis, dactylitis, and acute chest syndrome. Other acute complications include pneumonia, meningitis, sepsis and osteomyelitis, stroke, avascular necrosis, priapism, and venous thromboembolism.(8) Fuggle et. al., examined the severity and frequency of sickle cell related pain. They found that pain usually occurs once every 14 days, and pain ratings revealed that it was significantly more painful in children with SCD than it was for controls.(9) High rates of disruption are brought on when one is unable to engage in hobbies, play sports, or visit with friends.(9) A study done in Nigeria among students with sickle cell disease informed that absence from school of greater than one week was associated with poor performance, and children with sickle cell anemia had higher rates of school absence.(10) Academic absenteeism may hinder adolescents with SCD from receiving appropriate specialized educational interventions and may result in lower academic attainment.(11) According to a study conducted in Saudi Arabia on university, middle school, and high school children with SCD, 43 percent of the participants missed more than 14 days of school annually, compared to just 11 percent of the healthy participants, during the 2019 academic year.(12) Another study found that 68% of patients with SCD reported missing at least one important exam each year, and 85% of adult SCD patients reported missing school on average once per week.(13) Schwartz and others hypothesized the associates of school absenteeism with sickle cell disease.(14) Their study informs us that demographics, health related and psychosocial variables should be captured in subsequent studies. They found out that health-related and psychosocial variables were related to absenteeism, but demographic variables were not. However, their study did not include students with SCD at tertiary institutions.

In transitioning to university life, students find themselves in a new environment, with demanding new domestic responsibilities and engaging in academic tasks that are perceived as different and are closely related to future success and dealing with their social life.(15) The findings of this study shed light on the multifaceted challenges these students face and the tailored interventions to support their well-being and better quality of life.

## Methods

This study aimed to explore the lived experiences of university students with sickle cell disease at Makerere University. A qualitative cross sectional study design was employed. In-depth interviews were done with adult university students living with SCD to explore their experiences within the university setting.

### Participant Recruitment

Purposive sampling was used to recruit participants for the study. Eligibility criteria included being 18 years or older, having a self-reported diagnosis of SCD, and being an undergraduate student at Makerere University. Those with SCD and another non-SCD related chronic disease were excluded. To increase awareness and reach potential participants, flyers were distributed at adult sickle cell clinics, and advertisements were posted on social media platforms. Additionally, sickle cell organizations were contacted to help promote the study and encourage participation.

### Data Collection

Interviews were conducted between 20th August, 2023 and 30th March, 2024, with each session lasting approximately 90 minutes. Two female researchers, V.N. and A.A, who were medical students, conducted the interviews with each participant separately. The qualitative data was gathered using an in-depth, semi-structured interview guide that was grounded in the literature and was pilot tested. The development of the interview schedule was a reflective process, considering the sensitive nature of the questions and their potential impact on participants’ lives.(16) Probes were used throughout the interviews to enrich responses, particularly in instances where participants struggled to understand the questions or elaborate on their answers. The questions were open-ended, designed not to elicit right or wrong answers, but rather to encourage a descriptive and reflective discussion. Data saturation was reached at participant 15.

To ensure accuracy and prevent data loss or recall bias, the interviews were audio-recorded and notes were taken. This allowed for capturing the exact responses of participants, including the nuances in the researchers’ reactions. The recordings also facilitated a more thorough understanding of the responses by allowing for pauses and playback as needed. All interviews were transcribed verbatim for detailed analysis.

#### Data Analysis

The transcripts were thoroughly cleaned, followed by an iterative process of reading and re-reading to develop codes and themes. These themes were then discussed and refined during team meetings to ensure a comprehensive understanding of the data (V.N, A.N, K.T,M and A.A).

We analyzed data thematically using Interpretative Phenomenological Analysis (IPA). This approach is ideal for small, homogeneous samples where participants are purposely selected for their shared experience of the phenomenon.(17) Each participant provided an in-depth, reflective narrative of their experiences from a personal perspective. We used a two-stage interpretation process known as ‘double hermeneutics,’ where participants first interpret their own experiences, and the researcher then interprets these accounts to gain a deeper understanding of the participants’ lived experiences.(18)

## Results

### General characteristics

A total of 15 individuals participated in the study. The average age of participants was 23 years (range 19–26), and the majority were male, comprising 60% (9) of the sample. The general characteristics of participants, including age, year of study, sex, and field of study, are summarized in Table 1.

This study explored the lived experiences of university students living with Sickle Cell Disease (SCD) using Interpretative Phenomenological Analysis (IPA). The analysis revealed several overarching themes and sub-themes that reflect the complexities of balancing social and academic life and health challenges.

### Overview of the themes.

Six themes were identified as being central to the experiences of university students living with SCD

## Theme 1: Academic Disruptions

### Subtheme 1: Educational Setbacks

Students experienced significant loss of time in their academic progress due to SCD-related health crises.

“I missed exams in my first year and lost a full year because of a crisis. I had to redo everything.”

(Student 9)

“When I got Avascular Necrosis, it got to stage three, and I had to drop out of school because I couldn’t do anything. I had to take a “dead year” to undergo surgeries.”

(Student 7)

### Subtheme 2: SCD Complications

Several students recounted how complications from SCD led to delays and prolonged hospitalizations, further disrupting their studies.

“Only with the difficulty caused by the disease, like having to walk long distances from my hall of residence to the college, it all becomes tough for me. Especially with my hip issue.”

(Student 2)

“Sometimes the pain is too much and I can’t do much. I just have to forgo class. […] If I get sick within the semester, I can miss like 1–2 weeks of lectures.”

(Student 4)

“There is a semester where I had bone pain all day and was dizzy. I was hospitalized and had gallstones and leg ulcers.”

(Student 11)

“This was last month, and it was because I had a severe thrombotic crisis that rendered me unable to walk, hence not being able to attend lectures for two weeks.”

(Student 12)

“It is especially challenging for me in my menstrual periods. I often have crises with hospital admissions during such days. During exam periods, I sometimes miss tests and lectures as a result. It is especially difficult while at school.”

(Student 6)

These complications resulted in significant academic setbacks, with participants missing crucial lectures and exams.

## Theme 2: Experience of Stigmatization

### Subtheme 3: Stigma and Its Emotional Impact

Participants described how stigmatization by peers added to the emotional burden of living with SCD.

“There was a day I came from class, and on returning to the hostel, I found a group of a few girls in the corridor holding my medication and talking about me. They were saying things like, ‘You mean she also has sickle cell disease?’ and they were busy laughing. It was so stigmatizing and depressing, especially since some of them were my friends and even my roommate.”

(Student 3)

This experience highlights the deep emotional impact of stigma, especially when it comes from people who are expected to be supportive.

“Let’s say if there was a possibility whereby I didn’t have the crutches and I was just a normal sickle cell person, people would not even know. […] But with the crutch, obviously everyone wonders how a young boy can be walking with a crutch. So everyone can look at the crutch and look at you, but of course, I just.. I just ignore it.”

(Student 1)

### Subtheme 4: Empowerment Through Disclosure and Advocacy

In response to such stigmatization, some participants chose to openly disclose their condition, which led to positive outcomes.

“To deal with the stigma, I decided to tell them that I had SCD. Everyone got to know, and I even started a campaign where all the warriors felt free to unveil their status. This helped us live freely with the rest of the students in the school.”

(Student 3)

“[…] I told her I’ve lived with sickle cell for 26 years. And you know, everyday I take tabs, but you don’t ask. But I know you go out and talk about it. So today I’m telling it to you. Not everybody with sickle cell disease has yellow eyes, not all of them, ask me. Some people are healthy, they’re doing well because you know, they are following procedures. […] I started talking to my friends, and I started the awareness campaign.”

(Student 5)

This proactive approach not only mitigated the effects of stigma but also fostered a sense of community and support among students with SCD.

## Theme 3: Support Systems

### Subtheme 5: Family Support

The unwavering support from family members emerged as a vital resource for students coping with SCD.

“I get all the support from my family members. My family has really been there for me; I’m so proud of them.”

(Student 9)

“For psychological support my mum has played a big role because she has supported me in all possible ways, and has been there for me.”

(Student 2)

“So any time in the holidays when I get an attack, […] they help carry me to the car, because then I am in a lot of pain, I can’t even move. They carry me, they be with me in the hospital, they give me company until I am actually discharged.”

(Student 1 on support during a painful crisis)

The reliability of family support provided students with a crucial safety net, particularly during health crises.

### Subtheme 6: Friendships and Social Support

Friendships also played a critical role in helping students manage the challenges of SCD while away from home.

“Specifically my classmates have been good to me, and I know they’ll continue to be good. […] My discussion group members have as well been really good to me, they adjust their schedules sometimes to accommodate me, and that’s how I get support.”

(Student 2)

“For me, it’s very good to make friends in our area because you know, anything can happen when you’re out of home. So you need to involve your friends so that in case anything happens, they know what to do and have one or two contacts of your family members.”

(Student 10)

This sense of preparedness and the involvement of friends provided an additional layer of security for the students.

### Subtheme 6: Need for Enhanced Health and Support Services

Students emphasized the need for improved and accessible health support services, including free sickle cell testing, genetic counseling, and medication specific to their condition. They also advocated for regular medical checks, such as routine CBC tests, and practical accommodations like ground-floor rooms in residence halls to ease mobility.

“The university can make arrangements and put up a facility at the university hospital, sensitize the warriors to get their medication from there and for the simple tests like CBC, since we need to have them routinely. They could put in place measures to address some challenges: say in case you are anemic, they could give you at least one unit of blood. For the halls of residence, I am lucky enough to be on the ground floor. But next year we must apply for change of rooms. If they could reserve a few ground floor rooms for students with sickle cells, in order to avoid climbing up and down the stairs.”

(Student 2)

“Because sickle cell disease can affect many sicklers’ mobility, so they can put, like a way, whereby if I am coming from outside on a boda boda, I don’t have to walk.”

(Student 1)

A boda boda is a bicycle or motorcycle taxi used in East Africa to transport passengers or goods.

“Like to have awareness campaigns, and try and advise people to test and to donate blood. Free testing and counseling services should be provided. Then they should also engage in community outreaches for those people living with sickle cell. So if I go missing at school, somebody from campus is able to locate me and you know, try and talk to me and see how I’m doing. Something like that.”

(Student 3)

“They should try and see how warriors also get free medication. Because I know we have a hospital here, right? But it is not equipped with all the medicine that warriors need.”

(Student 6)

### Subtheme 7: Reliance on the Students’ with Disability Scheme

Students with disability from SCD complications such as avascular necrosis benefit from the university’s existing disability support system, which provides them with personal helpers for tasks.

“I have a helper, because I’m on the disability scheme. So I have a helper who can sometimes help me carry my bag. When I want to send someone to those long distances, I can send that person. Sometimes if he’s with me, I can tell him to help me carry the seat to my class or where I’m going to sit.”

(Student 1)

## Theme 4: Effective Self-Management and Coping Strategies

### Subtheme 8: Managing Academic Stress and Health Challenges

Participants developed various strategies to manage the dual pressures of academics and health. They emphasize self-care routines, such as avoiding stress, taking medication consistently, eating adequately, and preparing for anxiety-inducing situations like exams by using painkillers and staying warm.

“As a student, I try to avoid pressure because you know, there is pressure that comes from campus. So I try to avoid that pressure. I also try to stay away from stress factors. Then I tried to eat once in a while. Yes. And take fresh juice, […] Then they make sure I take my meds every day on time, as I try to communicate with my doctors in case there is something that I’m not seeing right, happening.”

(Student 9)

“Every morning before I go for practicals or if I had an exam, I would first take a painkiller because I know there’s that anxiety. I always have a scarf—some examination halls don’t allow sweaters, but at least they accept a scarf. So I made sure I had a scarf around my neck, took my painkillers, and tried to take water.”

(Student 14)

This routine helped the student manage anxiety and prevent health crises during stressful academic periods.

### Subtheme 9: Stability Through Routine

Establishing and adhering to a consistent routine was another coping mechanism.

“What helps is taking pain medication three days before and three days after my period(menstrual periods). When I stick to this routine, everything is really okay.”

(Student 6)

This methodical approach to managing health challenges allowed participants to maintain stability in their daily activities and academic responsibilities.

## Theme 5: Feeling of Missing Out

Initial expectations of a lively university social life shifted due to the reality of managing their health condition.

“Before joining university, I used to say that the minute I enter university I will party hard, go to clubs but when I reached here, I got a bit challenged as you know living with the disease. I was afraid I would cause a crisis to myself which would take me about a month or more to recover.”

(Student 2)

## Theme 6: Being Normal

Despite the challenges that come with SCD, participants expressed a strong desire to engage fully in university life and participate in activities alongside their peers.

“When I’m in school, I’m doing what every normal person is doing. I always tell myself that I may have sickle cell, but sickle cell doesn’t have me. So when I’m in school, I am literally involved in everything.”

(Student 15)

“If you live stress free and you avoid the triggers, you can just actually be.. totally normal like.. apart from if your eyes also become yellow, some people can know. but not everyone knows. They’ll think you have yellow fever, and what. But you can actually just live a normal life.”

(Student 1)

This determination to participate in school life, while managing their condition, underscores the resilience of these students.

These findings illustrate the interaction between health challenges and academic life for university students living with SCD. The identified themes and subthemes offer insights into how these students navigate their educational journeys. They emphasize the importance of support systems, self-management strategies, and the pursuit of normalcy despite the constraints of their condition.

## Discussion

The lived experiences of university students with sickle cell disease (SCD) show the barriers, facilitators, and coping strategies that significantly affect their academic success and social integration.

The findings of this study are distinctive in that they explore the lived experiences of university students with SCD across different academic levels in the context of university life which is a more independent educational environment during emerging adulthood.(19) While prior research has examined the impact of the disease and the provider’s perspectives of transitioning adolescents and young adults with SCD,(5, 14, 20) our study addresses the experiences of students in this transitional phase within a low-income country. These findings offer recommendations for universities to tailor support systems that better address the needs of students with SCD to facilitate academic and social success during this pivotal stage of their education.

### Barriers to Academic Success and Social Integration

One of the most significant challenges we identified was the need for students with SCD to take breaks of absence from days to weeks and at severity, a “dead year” which is a temporary withdrawal from studies, due to severe health complications. These decisions, though necessary for health recovery, often lead to delays in academic progress and can contribute to feelings of frustration and discouragement. A participant described the onset of Osteonecrosis of the femoral head (ONFH) as a significant turning point in their academic journey. ONFH is a prevalent complication of SCD which occurs in 22% of patients. (21) As the condition progressed further, the physical limitations became overwhelming, forcing the participant to drop out of university. The severity of ONFH necessitated surgical intervention, leading to a “dead year” where they could not continue their studies. The impact of taking a dead year is profound, as it not only disrupts the students’ academic trajectory but also affects their sense of belonging and continuity with their peers. The necessity of taking these absences underscores the severity of SCD complications, such as frequent pain crises and hospitalizations, which can make it nearly impossible to maintain regular academic schedules. This interruption highlights the profound impact that serious health complications associated with sickle cell disease can have on educational attainment, requiring students to prioritize their health over academic progression.

Participants highlighted that the stress related to exams and academic pressures often triggers painful crises. Many reported that the anxiety they experience during exams can sometimes lead to a painful episode, particularly a vaso-occlusive crisis (VOC). This observation aligns with existing research suggesting that mental stress can induce a VOC in individuals with SCD due to neural-mediated vasoconstriction.(22) The link between psychological stress and physical health in SCD underscores the importance of addressing both the academic and mental health needs of these students. Implementing strategies to reduce stress, especially during exam periods, may help lower the frequency of VOCs and support better academic outcomes for students with SCD.

### Social Integration and Stigma

After joining the university, students with SCD often experience a sense of “missing out” on social activities such as partying and dating. The physical limitations imposed by the disease, such as the need to avoid strenuous activities and late-night events may result in social isolation. Students described fear of triggering pain attacks and the potential need to disclose their sickle cell status often leads these students to withdraw from social interactions. This isolation is further compounded by physical limitations, such as the need to use crutches in cases of common SCD orthopedic complications such as ONFH, which not only hampers mobility but also restricts participation in physical activities. This finding is in line with prior work that explained that children and adults with SCD are at risk of disability which may lead to activity limitations and restricted social participation.(23)

Furthermore, the stigma associated with SCD was a recurrent theme, with some students experiencing negative attitudes and misconceptions from their peers. Previous research has documented similar experiences among individuals with SCD(24, 25) and chronic illnesses.(26) Students recounted encounters with gossip and ridicule from peers, especially when their medication was discovered, which in turn led to feelings of depression and isolation. These experiences highlight the pervasive impact of stigma on both the mental health and social life of university students with SCD. These results were consistent with previous studies that have shown that stigma has psychological and social consequences that can result in concealment as a coping strategy.(27) Visible signs of orthopedic complications of the disease, such as the use of crutches and limping in osteonecrosis of the femoral head, often attract unwanted attention and contribute to stigmatization.

### Facilitators to Academic Success and Social Integration

Despite these challenges, several facilitators were identified that helped students manage their condition and achieve academic success. Self-awareness emerged as a crucial factor; students who understood their triggers, such as cold weather and stress, were better able to manage their disease and minimize attacks. Medication compliance also played a vital role, with students regularly using prescribed medications like folic acid and hydroxyurea to prevent crises.

A supportive environment was another key facilitator. Some students with disabilities as a result of ONFH benefited from having a helper (a personal assistant provided by the university under the government scholarship scheme for students with disability), which significantly aided their mobility and allowed them to participate more fully in academic and social activities. Our study also found that seeking assistance from family and university health services, forming close relationships with understanding peers, and utilizing personal resilience helped the students to persevere through difficult times.

### Coping Mechanisms

The study found that students with SCD employ various coping mechanisms to manage their condition while pursuing their education. Avoidance of known triggers was a common strategy among the students. Participants described avoiding triggers such as cold environments and managing stress levels. As a result, they were able to maintain their health and continue their studies with minimal disruptions.

The findings suggest that disclosure can be a powerful coping mechanism for students with SCD. A student recounted a moment when a friend, unaware of their condition, made assumptions about what people with SCD should look like—skinny, with yellow eyes, and generally weak. When the student revealed that they had lived with SCD for 26 years without displaying these visible traits, it prompted a conversation that helped dispel common misconceptions. This honest exchange not only corrected the friend’s misunderstandings but also motivated the student to start raising awareness more broadly. Through sharing their experience, students found that talking openly about SCD reduced stigma and encouraged others to ask questions which fostered a more supportive environment. Disclosure turns potential stigma into opportunities for education and advocacy.

Establishing personalized routines based on self-awareness of individual triggers and needs plays a crucial role in helping students with SCD navigate the less predictable university environment. For instance, students described taking painkillers before exams to manage anxiety-induced pain episodes, along with carrying a scarf to examination halls to stay warm in situations where sweaters were not permitted. This approach not only mitigated potential disruptions to their academic participation and enhanced their overall well-being. Other studies have similarly highlighted the importance of self-management strategies and tailored routines in improving the quality of life for individuals with chronic conditions.(28) Ensuring that students with SCD have the support to develop and maintain these routines could be key in fostering both their academic success and overall well-being in the university setting.

#### Desired Interventions

The students’ experiences point to the need for more comprehensive support systems at the university level. Creating awareness about SCD among the university community is crucial in fostering a more supportive environment for affected students. Awareness campaigns could be implemented through workshops, seminars, and social media initiatives, aimed at educating both faculty and students about the challenges faced by those with SCD. One suggested intervention is allowing boda-bodas (motorcycle taxis) to transport students directly to their destinations within the campus, reducing the physical strain of moving around. Establishing specific support groups for students with SCD would also provide a platform for sharing experiences and strategies for managing the condition. Additionally, the availability of free medications, such as hydroxyurea, would significantly alleviate the financial burden associated with managing SCD. These efforts would help reduce stigma and misconceptions, encourage empathy, and promote the development of tailored accommodations.

#### Implications for University Policy

Our study suggests that universities need to develop more comprehensive support systems for students with SCD. This includes not only academic accommodations, such as flexible deadlines and attendance policies, but also increased awareness among faculty and peers about the nature of SCD and its impact on students. Universities should consider implementing policies that allow for more seamless reintegration after a dead year, as well as offering counseling services to help students cope with the psychological impact of their health challenges. Addressing these issues could significantly improve the academic and social experiences of students with SCD, enabling them to achieve their educational goals without compromising their health.

#### Limitations and Future Research

This study is limited by the small sample size and the fact that participants were recruited from a single university. Future research should aim to include a larger and more diverse sample to capture a broader range of experiences. Additionally, longitudinal studies would be beneficial to track the long-term impact of SCD on academic progression and social integration, particularly in relation to the effects of taking a dead year and managing SCD complications over time.

## Conclusion

In conclusion, this study highlights the significant challenges faced by university students with SCD, particularly in relation to academic interruptions and social integration due to health complications. Despite these challenges, the resilience and coping strategies exhibited by these students underscore their determination to succeed in their academic pursuits. Universities must recognize the unique needs of this population and implement policies that provide adequate support to ensure that students with SCD can thrive both academically and socially.

## Supplementary Material

Supplementary Files

This is a list of supplementary files associated with this preprint. Click to download.

- TrueInterviewGuidefortheStudy.pdf

## Figures and Tables

**Table 1:** Participant characteristics

Patient Number	Field of study	AGE	YEAR OF STUDY	SEX
Student 1	Computer Science and Information Technology	19	1	M
Student 2	Veterinary Medicine and Animal Sciences	20	1	M
Student 3	Veterinary Medicine and Animal Sciences	24	4	F
Student 4	Humanities and Social Sciences	24	3	F
Student 5	Natural Sciences	26	4	F
Student 6	Health Sciences	24	3	F
Student 7	Health Sciences	23	4	M
Student 8	Health Sciences	22	3	F
Student 9	Law	22	3	M
Student 10	Education	24	3	F
Student 11	Health Sciences	25	4	M
Student 12	Natural Sciences	26	3	M
Student 13	Engineering and Technology	24	3	M
Student 14	Veterinary Medicine and Animal Sciences	23	3	M
Student 15	Natural Sciences	24	3	M

## Data Availability

All data generated or analysed during this study are included in this published article and its supplementary information files.

## Abbreviations

SCD: Sickle Cell Disease
IPA: Interpretative Phenomenological Analysis
ONFH: Osteonecrosis of the Femoral Head
VOC: Vaso-Occlusive Crisis
CBC: Complete Blood Count
REC: Research Ethics Committee
SPH: School of Public Health
WHO: World Health Organization
